# Respiratory Response of the Deep-Sea Amphipod *Stephonyx biscayensis* Indicates Bathymetric Range Limitation by Temperature and Hydrostatic Pressure

**DOI:** 10.1371/journal.pone.0028562

**Published:** 2011-12-09

**Authors:** Alastair Brown, Sven Thatje

**Affiliations:** University of Southampton, Ocean and Earth Science, National Oceanography Centre, Southampton, Southampton, United Kingdom; University of Hamburg, Germany

## Abstract

Depth zonation of fauna on continental margins is well documented. Whilst increasing hydrostatic pressure with depth has long been considered a factor contributing significantly to this pattern, discussion of the relative significance of decreasing temperature with depth has continued. This study investigates the physiological tolerances of fed and starved specimens of the bathyal lysianassoid amphipod *Stephonyx biscayensis* at varying temperature to acute pressure exposure by measuring the rate of oxygen consumption. Acclimation to atmospheric pressure is shown to have no significant interaction with temperature and/or pressure effects. Similarly, starvation is shown to have no significant effect on the interaction of temperature and pressure. Subsequently, the effect of pressure on respiration rate is revealed to be dependent on temperature: pressure equivalent to 2000 m depth was tolerated at 1 and 3°C; pressure equivalent to 2500 m depth was tolerated at 5.5°C; at 10°C pressure equivalent to 3000 m depth was tolerated. The variation in tolerance is consistent with the natural distribution range reported for this species. There are clear implications for hypotheses relating to the observed phenomenon of a biodiversity bottleneck between 2000 and 3000 metres, and for the potential for bathymetric range shifts in response to global climate change.

## Introduction

The deep sea is one of the largest habitats on Earth. Phenotypic [1], [2] and genetic [3] clines with depth are evident in deep-sea organisms, which appear biochemically adapted to specific depth regimes [4], [5]. A compilation of 34 regional case histories and additional studies by Carney [6] indicates that deep-sea zonation patterns are widespread, with continental slope fauna clearly distinct from shelf fauna above and abyssal plain fauna below. High species turnover consistently indicates biodiversity bottlenecks at a shelf-slope transition between the shelf break and 1000 m, and a slope-abyss transition between 2000 and 3000 m. Subsequently, considerable research has focused on the physiological constraints to species bathymetric distributions (reviewed by [6], [7]).

Hydrostatic pressure (0.1 MPa = 10 m depth) effects on living systems initially result from thermodynamic shifts in chemical reaction rates [7]. Significant effects of hydrostatic pressure have been shown by pressure research on isolated biochemical systems, focusing on enzymatic proteins and lipoprotein membranes [6]. Denaturation of proteins resulting from pressure induced conformational change is well known [7] and adaptation to the high pressure and low temperature conditions prevailing in the deep sea is required [4], [5]. Temperature also acts as a thermodynamic parameter with decreasing temperature decreasing chemical reaction rates; rates change by a factor of two to three for each 10°C temperature change [6]. Successful adaptation to low temperature and high pressure habitats involves increased enzyme concentration, adoption of enzymes with greater efficacy and inclusion of modulator compounds that facilitate enzyme reactions ([8]–[11] and references cited therein). Increased hydrostatic pressure and decreased temperature also reduce the fluidity of bio-membranes necessitating homeoviscous adaptations in membrane structure and composition [12], [13]. In the absence of these adaptations, the effects of high pressure and low temperature are sufficient to affect biological processes at all levels of organisation [7], [9]. These effects appear to contribute to limited thermal tolerance through oxygen-limitation, i.e. by reducing oxygen supply (for review see [14]). Given the analogous effects of low temperature and high pressure it has recently been proposed that pressure effects on oxygen supply similarly determine tolerance to pressure (e.g. [15]). Since respiratory rate has previously been used as an indicator of metabolic rate in marine ectotherms (see [14]) decreases in oxygen consumption are suggested to indicate decreasing metabolism resulting from reduced oxygen supply, reflecting an inability to tolerate exposure conditions. Anaerobic processes are not stable over time as the product (lactate) requires subsequent oxidation before removal as CO_2_ and water [16], and consequently survival under these conditions is time limited [14].

To the authors knowledge no organism-level study has extensively examined the interaction of hydrostatic pressure and temperature effects on a deep-sea species across and beyond the entire range experienced within the known natural distribution. This study tests the hypothesis that the bathymetric range of the necrophagous bathyal amphipod species *Stephonyx biscayensis* (Chevreux, 1908) is constrained by temperature and hydrostatic pressure. This species is common at depths between 500 and 2000 m across the North-East Atlantic Ocean from Iceland to the Cape Verde Islands (M.H. Thurston, personal communication) and has also been reported from 494 m depth in the Gulf of Mexico and from 549 to 900 m in the Caribbean Sea [17]. In light of the food storage behaviour of other necrophagous lysianassoid amphipod species [18] and the potential for considerable variation in respiration rate with time elapsed since the consumption of food, the effect of starvation on respiration rates of specimens exposed to these thermal and hyperbaric conditions is also investigated. Results indicate that the pressure tolerance of this species is temperature dependent and are discussed with respect to the depth tolerance of this species, the phenomenon of a biodiversity bottleneck between 2000 and 3000 m, and the potential for bathymetric range responses to ocean warming.

## Materials and Methods

All experiments were conducted in accordance with the legal requirements of the United Kingdom. The use of Crustacea is unregulated in the United Kingdom and subsequently does not require ethics approval by a specific committee.

### Sampling and maintenance

**Figure 1:**
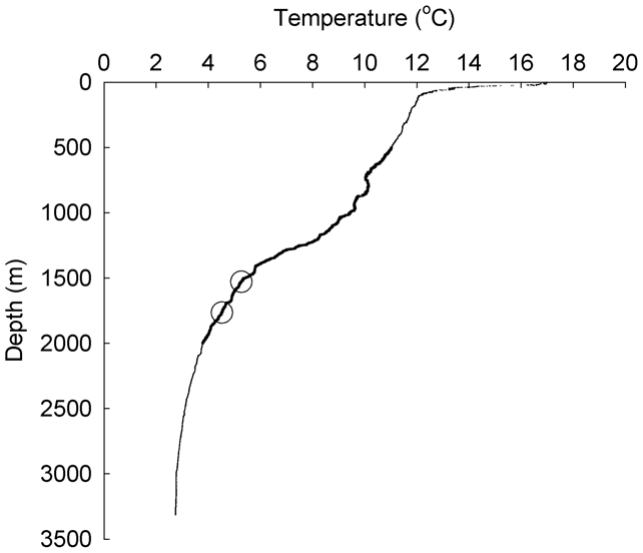
Temperature at depth in the Whittard submarine canyon, Gulf of Biscay. Temperature was sampled by ROV on 17^th^ July 2009. Temperatures within the known bathymetric range of the lysianassoid amphipod *Stephonyx biscayensis* are indicated by a heavier line. Depths of amphipod trap deployments and the corresponding temperatures are indicated by open circles.

Adult specimens of the scavenging amphipod species *Stephonyx biscayensis* were collected on 29^th^ June and 23^rd^ July 2009 from depths of 1528 m (48°53.84N, 11°08.36W) and 1765 m (48°23.81N, 10°18.99W) in the Whittard submarine canyon, using a baited (mackerel) trap deployed from RRS James Cook on cruise JC36 [19]. Animals had direct access to the bait and the trap was neither insulated or pressure retaining. No mortality was observed based on several hundred specimens retrieved from the trap and on subsequent days. Animals were maintained at atmospheric pressure, ambient salinity (35.2), and temperature of 5.5°C (the temperature at the first trap site; Fig. 1), and 24-h darkness. Upon return to the National Oceanography Centre, Southampton, animals were transferred to a recirculating aquarium at atmospheric pressure, ambient salinity (32.7), temperature of 8°C (the temperature at the mid-depth of the species' bathymetric distribution; Fig. 1), and 24-h darkness.

### Respiration rate

Oxygen consumption rates (MO_2_) of individual animals were used to assess acute respiratory response to pressure following a minimum of 3 hours exposure to experimental temperatures. Experimentation was conducted within 1 week of capture (unacclimated to atmospheric pressure and fed), after 2 months food deprivation (acclimated to atmospheric pressure and starved), and within 1 week of feeding (cod) subsequent to 2 months food deprivation (acclimated to atmospheric pressure and fed). Experimental pressures (0.1, 5, 10, 15, 20, 25 or 30 MPa) and temperatures (1, 3, 5.5 or 10°C) were selected to represent those found across and beyond the natural distribution of the species. Due to cruise time constraints experiments with unacclimated animals were restricted to the two temperatures closest to that of the sample site: i.e. 3 and 5.5°C. Five individuals were exposed to each experimental combination. Animals were sampled randomly and were not reused in the experiments; each specimen was exposed to a single combination of experimental conditions. A total of 70 unacclimated and fed animals, 140 acclimated and starved animals, and 140 acclimated and fed animals were used in experimental treatments.

Oxygen consumption rates were measured using an adaptation of previously described protocols [20], [21]. In brief, an individual was isolated in a 2.5 ml transparent plastic vial filled with oxygen saturated, filtered seawater. The vial was closed underwater to ensure the absence of air bubbles and was placed inside a temperature acclimated experimental pressure vessel (see Fig. 1 in [22]) filled with previously incubated freshwater. Pressurisation of the experimental vessel was continuous and acute, taking less than 10 seconds, and was achieved using a Maximator M72 manual air-driven liquid pump. The pressure vessel was submerged in a water bath controlled by a Haake EK20 chiller and a Haake DC10 heater to maintain constant temperature. After 10 minutes the experimental vessel was removed from the water bath and the pressure was released instantaneously. The plastic vial was inverted three times to ensure that the oxygen concentration of the seawater was homogenous. The lid was carefully removed to avoid spilling water and the percentage oxygen saturation of the water in the vial was assessed using an oxygen microoptode connected to a Microx TX3 array (PreSens, Germany); oxygen measurements were made within 45 seconds of depressurisation. A single animal was used per incubation. Individuals were not restrained by the vials and displayed no immediate behavioural response to isolation in the vial, i.e. animals remained immobile. Subsequent to enclosure of the vial in the pressure chamber no observation of animal behaviour was possible.

To eliminate any bias due to bacterial oxygen demand or calibration, individual respiration rates (MO_2_) were obtained by comparison with control chambers (no animals) exposed to the same experimental conditions. Respiration rates are therefore an average for the 10 minutes during which the organisms were exposed to experimental conditions. Oxygen saturation did not fall below 50% saturation under any treatment, minimising the potential for any hypoxic exposure effect [23]. All specimens survived experimental treatment.

The absence of correlation between body size and weight-specific oxygen consumption has been reported for deep-sea amphipods and attributed to species-specific metabolic characteristics and adaptation to low food supply [24]. Subsequently, no restriction of weight range or correction for body weight was made. General linear model ANOVA of wet masses of animals under each experimental treatment indicated no significant differences (*F*_69,280_ = 1.32, *p* = 0.063), i.e. there was no significant potential for size-scaling respiration effects between treatments. Although discussion continues, it has been concluded previously that serious errors of interpretation concerning whole animal rates can result from using forms of standardisation other than wet mass (e.g. [25]). Additionally, rates of oxygen consumption available for comparison in pressure literature are predominantly derived using wet mass (e.g. [20], [21], [24]–[27]). Total wet mass of animals was determined to the nearest 0.01 mg after thorough blotting, before animals were returned to the aquarium. Wet mass ranged from 67.80 mg to 297.63 mg. Individual respiration rates were expressed as µmol O_2_ g^−1^ h^−1^.

Data were not normally distributed (Kolmogorov-Smirnov test, *p*<0.05) however “the assumption that data are normally distributed is not very important…The analysis of variance is quite robust to non-normality – in other words, its outcome and interpretation are not affected by the data being non-normal…this is particularly the case where experiments are large (there are many treatments) and/or samples of each treatment are large. It is also the case where samples are balanced” ([28], 194). “Only very skewed distributions would have a marked effect on the significance level of the *F*-test or on the efficiency of the design” ([29], 407). In the present study there are many treatments, the samples are balanced, and the distributions were not very skewed. In contrast the consequences of heterogeneous variances are significant (see e.g. [28], 181–3). Square root transformation was necessary to achieve homoscedasticity (Levene's test, *p*>0.05). General linear model three-way ANOVA was used to compare respiration rates under different combinations of factors. Where no significant interaction is reported between three factors, this method enables examination of interactions between pairs of factors by pooling all levels of the third factor, and without conducting additional tests [28], [29]. Subsequently, where no significant interaction is reported between pairs of factors, further pooling is used to examine the effect of individual factors, again without additional tests [28], [29]. The post-hoc multiple comparisons Holm-Sidak test was used to determine which treatments produced differences [29].

## Results

### Acclimation, temperature and pressure effects on respiration rate

**Figure 2:**
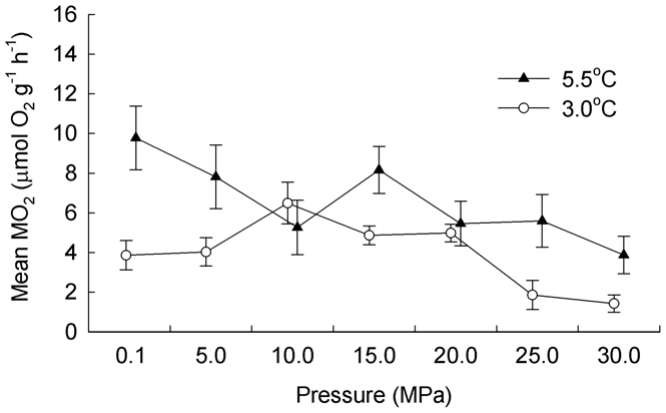
Acclimation, temperature and pressure effects on the rate of oxygen consumption of *Stephonyx biscayensis*. In the absence of an effect of acclimation to atmospheric pressure on the interaction of temperature and pressure effects, unacclimated and acclimated treatments were pooled. The effect of temperature on the relationship between pressure and mean (±1 s.e.) rate of oxygen consumption (MO_2_) of fed *Stephonyx biscayensis* is shown (10 individuals per data point); treatments are offset for clarity and no treatments differ significantly from 0.1 MPa.

Comparing mean MO_2_ of unacclimated and fed animals to mean MO_2_ of acclimated and fed animals (3 and 5.5°C only; *n* = 140) indicated that acclimation had no significant effect on the relationship between the effects of temperature and pressure (*F*_6,112_ = 1.58, *p* = 0.159). The effect of pressure was dependent on temperature (Fig. 2; *F*_6,112_ = 2.41, *p* = 0.032). However, no treatments were significantly different relative to the MO_2_ at atmospheric pressure. Both the effect of pressure and the effect of temperature were independent of acclimation (*F*_6,112_ = 1.17, *p* = 0.325 and *F*_1,112_ = 0.31, *p* = 0.589 respectively). The effect of acclimation as an individual factor, when all levels of both other factors were pooled, was to significantly increase MO_2_ by 18% (*F*_1,112_ = 6.52, *p* = 0.012).

### Starvation, temperature and pressure effects on respiration rate

**Figure 3:**
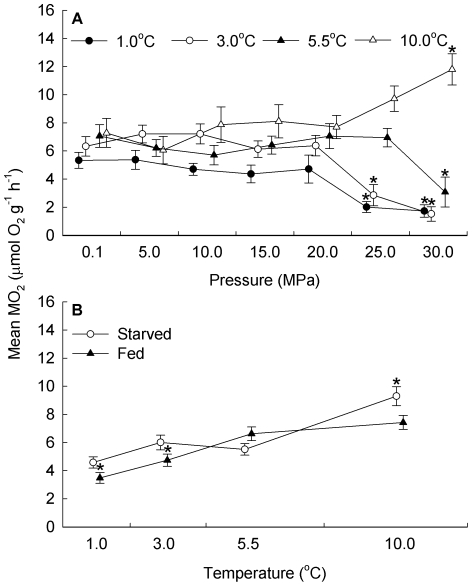
Starvation, temperature and pressure effects on the rate of oxygen consumption of *Stephonyx biscayensis*. (**A**) Fed and starved treatments were pooled in the absence of a starvation effect on the interaction of temperature and pressure effects. Subsequently the effect of temperature on the relationship between pressure and mean (±1 s.e.) rate of oxygen consumption (MO_2_) of *Stephonyx biscayensis* acclimated to atmospheric pressure is shown (10 individuals per data point). (**B**) Similarly, pressure treatments were pooled in the absence of a pressure effect on the interaction of starvation and temperature effects. Subsequently the effect of starvation on the relationship between temperature and mean (±1 s.e.) MO_2_ of *Stephonyx biscayensis* acclimated to atmospheric pressure is shown (35 individuals per data point). Treatments are offset for clarity and those differing significantly from 0.1 MPa (**A**) or 5.5°C (**B**) are indicated by an asterisk.

Comparing mean MO_2_ of acclimated and starved animals to mean MO_2_ of acclimated and fed animals (*n* = 280) indicated that starvation had no significant effect on the relationship between the effects of temperature and pressure (*F*_18,224_ = 1.32, *p* = 0.176). The effect of pressure was temperature dependent (Fig. 3A; *F*_18,224_ = 7.31, *p*<0.001). MO_2_ of animals at 1°C remained relatively stable from 0.1 MPa to 20 MPa before decreasing significantly at 25 MPa. Similarly, MO_2_ of animals at 3°C remained relatively stable from 0.1 MPa to 20 MPa before decreasing significantly at 25 MPa. In contrast, MO_2_ of animals at 5.5°C remained relatively stable from 0.1 MPa to 25 MPa before decreasing significantly at 30 MPa. Further, MO_2_ of animals at 10°C remained relatively stable from 0.1 MPa to 25 MPa before increasing significantly at 30 MPa.

The effect of pressure was independent of starvation (*F*_6,224_ = 1.34, *p* = 0.241). However, the effect of temperature depended on starvation (Fig. 3B; *F*_3,224_ = 5.29, *p* = 0.002). The MO_2_ of starved animals remained relatively stable from 1°C to 3°C and to 5.5°C before increasing significantly at 10°C. In contrast, the MO_2_ of fed animals increased significantly from 1°C to 3°C, increased significantly to 5.5°C, and remained relatively stable to 10°C.

## Discussion

### Methodological considerations

Measurement of acute respiratory response was both necessary as a result of technological constraints, and expedient in assessing the organism's ability to tolerate acute exposure to extreme environmental conditions. Long-term maintenance of organisms at high pressure is possible (reviewed by [7]). However, assessing the respiratory rate of small organisms in high-pressure aquaria is impractical. The approach taken in this study also avoids multiple acute pressure changes made in earlier investigations of respiratory response to hydrostatic pressure (e.g. [27]) and should thus increase confidence in results. Critically, observations from acute experimental exposure represent maximum responses that can be expected to these environmental conditions and therefore this method can be used to assess the tolerance ranges expected for the species (e.g. [20], [21]).

Although respiratory rates measured in acute exposures may be higher than routine rates [e.g. 27] they are internally consistent and thus allow comparison of treatment effects. It has been previously indicated that patterns of pressure effects are the same regardless of acclimation pressure [27]. The absence of acclimation interaction effect in this study is consistent with this, suggesting that although rates of individuals acclimated to atmospheric pressure may not accurately represent rates of those acclimated to *in situ* pressure, the patterns of starvation, temperature, and pressure effects reported here are representative of individuals acclimated to higher pressures. The absence of statistically significant decreases in respiration rate in pooled unacclimated/acclimated data may result from greater variance, attributable to a larger acclimation than starvation effect (18% and 12% respectively).

Respiration rate of the deep-sea mysid *Gnathophausia ingens* has been reported to decrease over time following acute pressure change (inferred from [27]). However, this reduction has been attributed to a transient increase in the activity of animals immediately following treatment, before activity declined to a more constant rate [27]. With the exception of pooled starved and fed treatments at 10°C and 30 MPa no significant increase in oxygen consumption is evident in the present study. Although it is possible that the duration of the experimental exposure may not be sufficient for observation of an activity response, the difference in the response of study species is likely to result from the difference in habits: *G. ingens* is bathypelagic whereas *S. biscayensis* is benthic. Subsequently, it is anticipated that little variation in mean respiration rate would be observed over time in *S. biscayensis*.

### Starvation, pressure and temperature effects on metabolic rate

As expected [11], respiration rates recorded during this study are within the range reported for other lysianassoid amphipod species at comparable temperatures (<0.1 to 25.8 µmol O_2_ g^−1^ h^−1^; see Table 5 in [24]), including rates measured *in situ* in the deep sea at 3°C (0.9 to 9.4 µmol O_2_ g^−1^ h^−1^; [30]). The standard deviations are also similar to those reported elsewhere for shallow-water lysianassoids at atmospheric pressure and under chronic exposure to hydrostatic pressure, and for other deep-sea crustaceans both at atmospheric and *in situ* pressure [25], [26], [30], [31]. The observed increase in respiration rate in response to starvation is consistent with effects reported for the Antarctic lysianassoid *Waldeckia obesa* [32] and is likely to result from the batch reactor digestion model and behavioural adaptation to food limitation reported for other deep-sea lysianassoids, e.g. decreased metabolic rates allow ingested and stored energy to support mature specimens of *Eurythenes gryllus* for an estimated 9–22 months [18], [33].

Effects of low temperatures and high pressures on physiological processes have previously been observed to be analogous [34]. *In vitro* evidence indicates that critical enzyme functionality can be maintained under different pressure and temperature regimes by changes in the amino acid sequence in the enzyme, or by the inclusion of stabilizing compounds in the intracellular milieu [5]. Accumulation of higher levels of lipid has also been observed, counteracting pressure and temperature induced decrease in membrane fluidity [35]. According to this model, the observed decreases in respiratory rates of *Stephonyx biscayensis* at high hydrostatic pressure and low temperature indicate decreased metabolism resulting from reduced oxygen supply in the absence of such adaptations. The absence of decrease in oxygen consumption rates at higher temperature indicates no reduction in oxygen supply, revealing that the elevated temperature enables tolerance of higher pressure, at least in the short term. This suggests that the effects of temperature and pressure are cumulative.

The combinations of temperature and hydrostatic pressure used during this study were chosen to represent the environmental conditions found in and around the natural distribution of this species. The levels at which physiological impairment occurs (hydrostatic pressure greater than 20 MPa at temperatures of 1°C and 3°C) are consistent with the limit of the natural distribution range reported for *S. biscayensis* and with conditions found at sampling sites in the Whittard submarine canyon (Fig. 1). This contrasts with the response of the amphipod *Eurythenes gryllus*, reported from surface layer depths of both polar regions to 7800 m in the Atacama Trench [36], which has been found to maintain approximately constant rates of oxygen consumption at hydrostatic pressures from 1 to 325 atm (1 atm = 0.1 MPa) at 2°C [37]. That the natural distribution of *S. biscayensis* coincides with the physiological impairment observed under acute experimental exposure to temperature and hydrostatic pressure suggests these factors may constrain the lower bathymetric limit of this species. Indeed, a congeneric species (*Stephonyx mytilus*) is reported from 2482 to 2635 m depth at Galapagos vent sites and East Pacific Rise 13°N vent sites [38] where temperatures are typically elevated above those of the surrounding deep sea. It does not appear that pressure exerts an influence on the upper bathymetric limit of *S. biscayensis*, however it is possible that temperatures higher than those examined (i.e. >10°C) may constrain the range of this species. Although not reported for *S. biscayensis*, polar emergence is documented in the panoceanic lysianassoid amphipod *Eurythenes gryllus* [36], suggesting that the upper bathymetric limit of some lysianassoid species may be defined by temperature. Other ecological factors may also influence the upper limits to species' distributions, e.g. bathymetric environmental gradients may foster biological interactions such as competition with and predation by species restricted to shallower water (see [39] and references cited therein).

Whilst temperature has long been regarded as the principal factor restricting the latitudinal distribution of marine invertebrates (for review see [14]), hydrostatic pressure has been seen as one of the key factors limiting the lower limits of species' depth distributions [4], [5]. The interaction of hydrostatic pressure and temperature effects identified in this and other studies (e.g. [20]–[22]) supports a significant role for temperature within depth adaptation models, and provides a clear indication that effects of high hydrostatic pressure and low temperature are combined. Identification of a consistent biodiversity bottleneck between 2000 to 3000 m (see [6]) lead us to hypothesise that the physiological constraints imposed by the combined effects of high hydrostatic pressure and low temperature contribute to the limitation of bathymetric distribution in many species, with passage to deeper water requiring further adaptation to the prevailing deep-sea conditions. This appears consistent with hypotheses of deep-sea colonisation by shallow water invertebrates, indicated by onshore-offshore evolutionary dynamics (see [40] and references cited therein) and the relatedness of extant shallow- and deep-water taxa (e.g. [41]). Recent studies have emphasised this bottleneck for other crustaceans in an evolutionary context [20], [42].

Increases in depth range have been observed in North Sea fishes in response to climate change [43], [44] and it has recently been proposed that shallow-water invertebrates may be able to penetrate greater depths as continental shelf waters warm [20]. Increases of between 0.5 and 1.0°C are predicted to occur at depths beyond 2000 metres in some parts of the northern hemisphere by 2100 (see Fig. 10.7 in [45]). Further increases in deep ocean temperature are predicted beyond 2100 since the ocean will eventually warm up reasonably uniformly by the amount of the global average surface temperature change [46]. Multi-model means of oceanic warming under the moderate greenhouse gas growth scenario predict this to be between 1 and 2°C across most of the oceans for the period 2080–2099 relative to the period 1980–1999 [45]. The interaction of temperature and hydrostatic pressure demonstrated in this study by the absence of physiological impairment at 25 MPa at 5.5°C suggests that projected increases in bathyal temperatures may facilitate penetration of deeper water by this species. Although the importance of latitudinal shifts on ecosystem dynamics has been stressed as migrations to higher latitudes follow shifts of climate envelopes [47], [48], little consideration has been made of the possible interactions that may result from bathymetric shifts in the ranges of individual species. However, to fully assess the potential of organisms to increase depth penetration future studies will need to elucidate pressure tolerances throughout the various critical stages in ontogeny, e.g. for crustaceans embryogenesis, larval/adult moult, feeding and mating, and in a range of taxa.
